# P-1040. Epidemiology and Risk Factors for Vascular Access-associated Infections in Japanese Patients with Chronic Kidney Disease and on Hemodialysis

**DOI:** 10.1093/ofid/ofaf695.1235

**Published:** 2026-01-11

**Authors:** Keita Morikane

**Affiliations:** Yamagata University Hospital, Yamagata, Yamagata, Japan

## Abstract

**Background:**

Patients with chronic kidney dysfunction and on hemodialysis are subject to various infectious disease, including vascular access-associated infections (VAIs). However, the magnitude of VAI is not well investigated since nationwide surveillance of VAI has not been well established worldwide, except in the United States. In Japan, our group established a voluntary VAI surveillance scheme, Dialysis Surveillance Network Japan (DSN-J). The purpose of this study is to describe the epidemiology and risk factors for VAI in Japan.

**Methods:**

Data collected through DSN-J from January 2008 to December 2023 were used. Incidence of VAI was calculated by the number of infection per 1,000 dialysis sessions. Profile of causative pathogens as well as potential risk factors including type of access, diabetes, indication of catheter use and seasonality were analyzed.

**Results:**

During the study period, 5,907,641 dialysis sessions were surveyed at 52 hospitals. 1,350 VAIs were observed, with an overall incidence of 0.23 per 1,000 sessions. The annual incidence steadily declined over time. The incidence of VAI caused by non-cuffed catheter (NCC) was 7.75 (663 VAIs in 85,525 sessions). This was significantly higher than that with other access type (0.05 for arteriovenous fistula, 0.10 for superficialization of brachial artery, 0.49 for arteriovenous graft, and 1.39 for cuffed catheter (CC), respectively). The dominant causative pathogen was methicillin-susceptible *Staphylococcus aureus* (306 VAIs), followed by methicillin-resistant *Staphylococcus aureus* (227). Diabetic patients had a significantly higher risk of VAI than non-diabetic patients when they were on dialysis with NCC (Relative risk[RR]:1.24, 95%CI:1.04-1.47), but not so when with CC (RR:0.78, 95%CI:0.60-1.01). NCC inserted at the femoral site had significantly higher risk of VAI compared to that at internal jugular site (RR 1.46, 95%CI: 1.22-1.74). The incidence of VAI in summer (May to October) was substantially higher than that in winter (November to April), especially in patients with arteriovenous fistula or CC.

**Conclusion:**

The overall incidence of VAI is low, however that in NCC was unacceptably high. The epidemiology and risk factors identified through this research would help better control VAIs in patients on hemodialysis.

**Disclosures:**

All Authors: No reported disclosures

